# Supplementary figures and images for: Knowledge of COVID-19 symptoms, transmission, and prevention: Evidence from health and demographic surveillance in Southern Mozambique
Source: PLOS Glob Public Health. 2023 Nov 1;3(11):e0002532. doi: 10.1371/journal.pgph.0002532 (PMC10619866; doi:10.1371/journal.pgph.0002532)

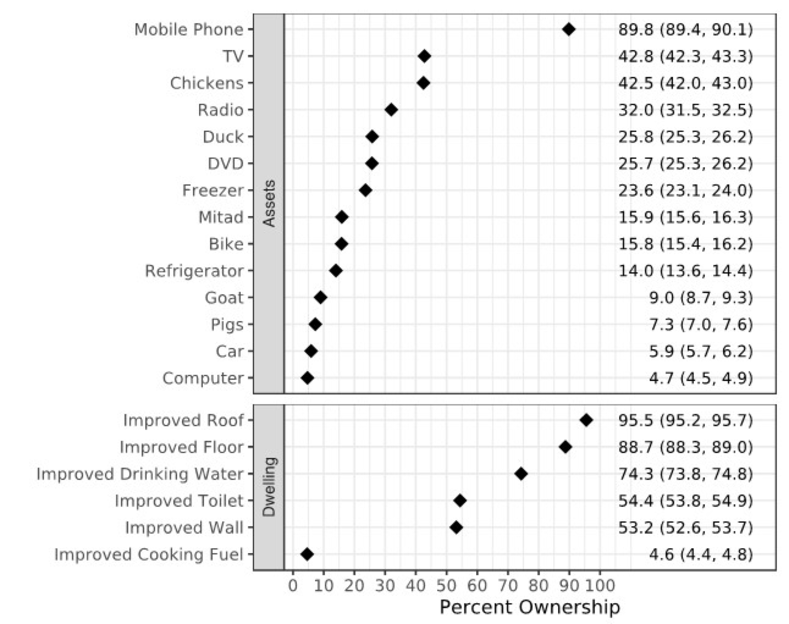

Supplement: S1 Fig — A Mitad is a portable, electric hotplate used for cooking. Improved classifications for dwelling materials were based on DHS recommendations. (TIF) [file pgph.0002532.s002.tif]

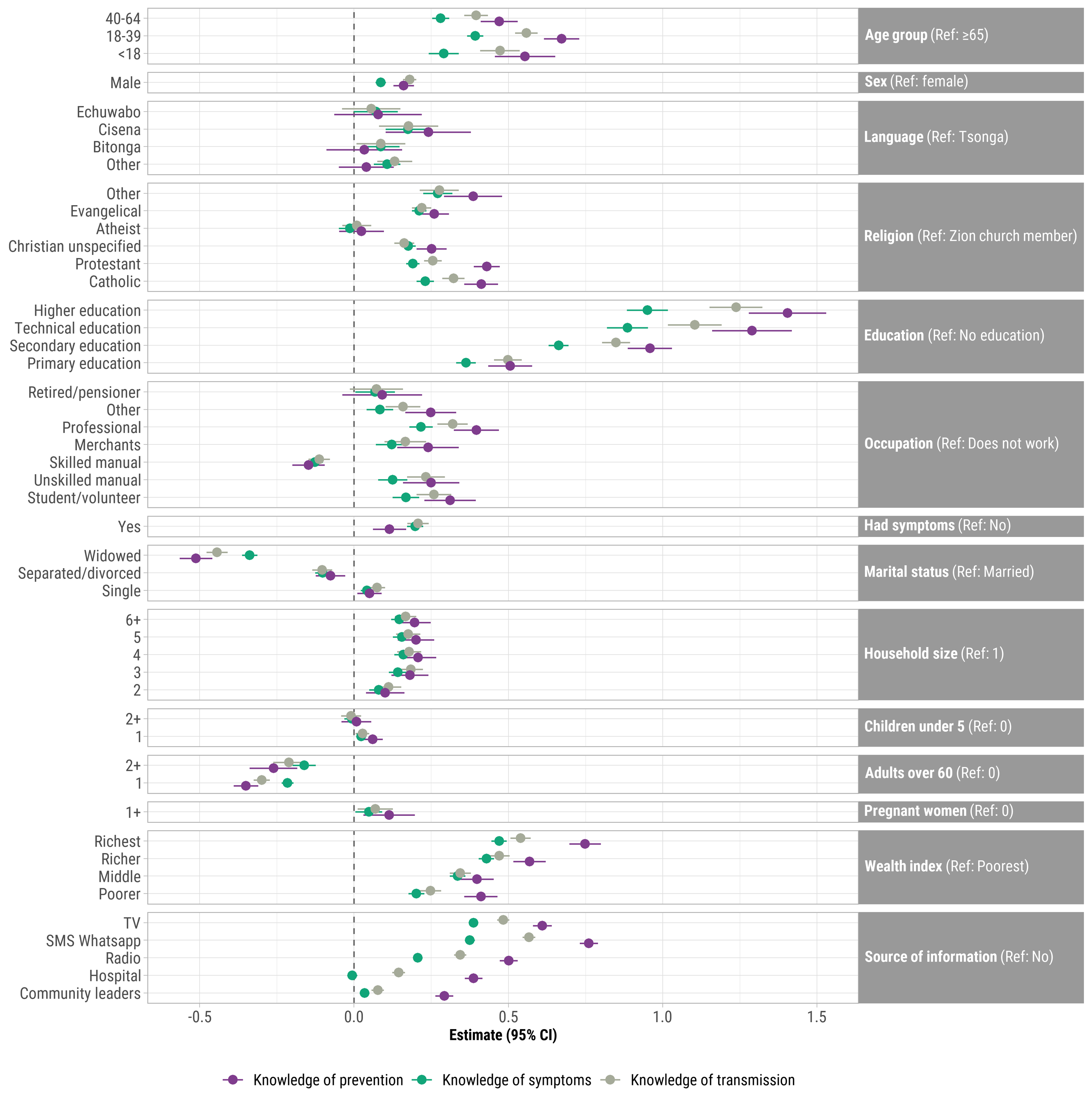

Supplement: S2 Fig — Points represent β coefficients and error bars represent 95% confidence intervals. Knowledge of prevention includes: Avoid crowded places, touching eyes, touching mouth, touching nose, or traveling; social distancing; quarantine; and wash hands with alcohol. Knowledge of symptoms includes: Difficulty breathing, dry cough, fever, headaches, muscle pain, and sore throat. Knowledge of transmission includes: Droplets from an infected person, hugging an infected person, kissing an infected person, touching a fomite, touching an infected person, touching an infected person’s hands, touching your eyes or nose, and touching your mouth. (TIF) [file pgph.0002532.s003.tif]

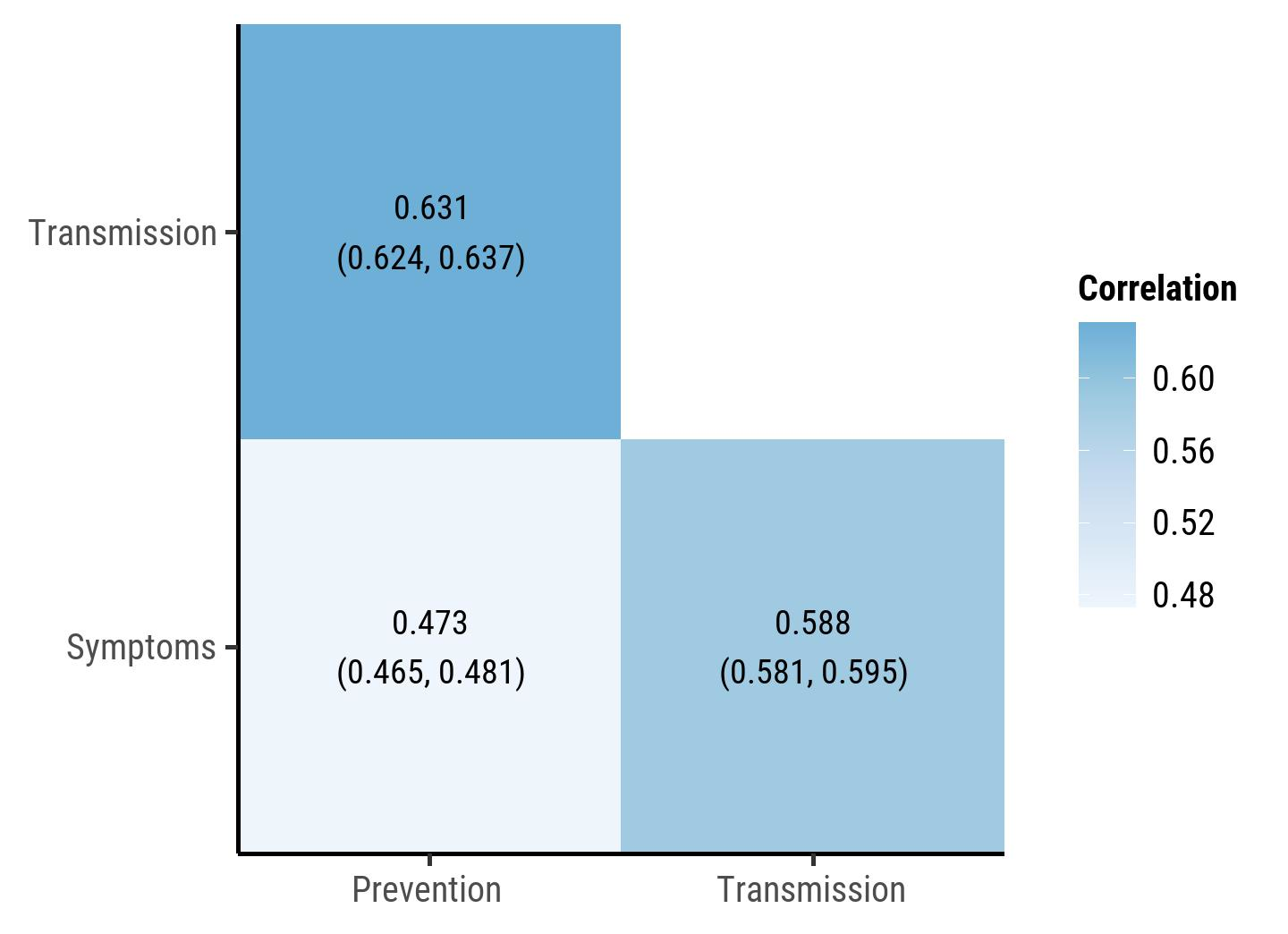

Supplement: S3 Fig — Coefficients (r) and 95% confidence intervals are shown. (TIF) [file pgph.0002532.s004.tif]
