# Supplementary material for: Knowledge of COVID-19 symptoms, transmission, and prevention: Evidence from health and demographic surveillance in Southern Mozambique
Source: PLOS Glob Public Health. 2023 Nov 1;3(11):e0002532. doi: 10.1371/journal.pgph.0002532 (PMC10619866; doi:10.1371/journal.pgph.0002532)
Supplement: S1 Table — (DOCX) [file pgph.0002532.s006.docx]

| S1 Table. Principal components factor analysis of knowledge variables, Mozambique, April 2021 – February 2022 (N=33,087). | | |
| --- | --- | --- |
| Characteristic | Factor Pattern |  |
| Knowledge of symptoms |  |  |
| Difficulty breathing | 0.55 |  |
| Dry cough | 0.61 |  |
| Fever | 0.58 |  |
| Headaches | 0.61 |  |
| Muscle pain | 0.39 |  |
| Sore throat | 0.60 |  |
| *Eigenvalue* | 1.89 |  |
| Explained variance | 32% |  |
| Knowledge of transmission |  |  |
| Droplets from an infected person | 0.44 |  |
| Hugging an infected person | 0.63 |  |
| Kissing an infected person | 0.62 |  |
| Touching a fomite | 0.41 |  |
| Touching an infected person | 0.45 |  |
| Touching an infected person’s hands | 0.49 |  |
| Touching your eyes or nose | 0.59 |  |
| Touching your mouth | 0.63 |  |
| *Eigenvalue* | 2.32 |  |
| Explained variance | 29% |  |
| Knowledge of prevention |  |  |
| Avoid crowded places | 0.46 |  |
| Avoid touching eyes | 0.81 |  |
| Avoid touching mouth | 0.80 |  |
| Avoid touching nose | 0.81 |  |
| Avoid traveling | 0.60 |  |
| Quarantine | 0.53 |  |
| Social distancing | 0.33 |  |
| Wash hands with alcohol always | 0.40 |  |
| *Eigenvalue* | 3.09 |  |
| Explained variance | 39% |  |
