# Supplementary material for: Knowledge of COVID-19 symptoms, transmission, and prevention: Evidence from health and demographic surveillance in Southern Mozambique
Source: PLOS Glob Public Health. 2023 Nov 1;3(11):e0002532. doi: 10.1371/journal.pgph.0002532 (PMC10619866; doi:10.1371/journal.pgph.0002532)
Supplement: S2 Table — (DOCX) [file pgph.0002532.s007.docx]

| Table S2. Descriptive statistics of individuals who participated in COVID-19 cross-sectional survey following multiple imputation, Mozambique, April 2021 – February 2022 (N=33,087) | |
| --- | --- |
|  | Imputed (N=33,087) |
| Characteristic | % |
| Individual characteristics |  |
| Age (years) (N=33,046) |  |
| <18 | 3.2 |
| 18-39 | 51.5 |
| 40-64 | 32.3 |
| ≥65 | 13.0 |
| Female sex (N=32,153) | 72.4 |
| Language (N=31,852) |  |
| Tsonga | 93.5 |
| Bitonga | 1.5 |
| Cisena | 1.0 |
| Echuwabo | 1.1 |
| Other | 2.9 |
| Religion (N=31,914) |  |
| Catholic | 8.7 |
| Protestant | 19.2 |
| Christian unspecified | 14.2 |
| Zion church member | 34.5 |
| Atheist | 6.2 |
| Evangelical | 14.7 |
| Other | 2.5 |
| Education (N=29,924) |  |
| No education | 10.3 |
| Primary education | 59.7 |
| Secondary education | 28.0 |
| Technical education | 1.0 |
| Higher education | 0.9 |
| Occupation (N=31,597) |  |
| Does not work | 9.7 |
| Student/volunteer | 3.7 |
| Unskilled manual | 2.9 |
| Skilled manual | 70.6 |
| Merchants | 2.4 |
| Professional | 5.3 |
| Retired/pensioner | 1.4 |
| Other | 3.8 |
| Marital status (N=31,891) |  |
| Single | 18.4 |
| Married/De facto union | 54.4 |
| Separated/divorced | 12.2 |
| Widowed | 15.0 |
| Had COVID-19 symptoms | 7.5 |
| Household characteristics |  |
| Household size (32,744) |  |
| 1 | 13.2 |
| 2 | 13.1 |
| 3 | 14.8 |
| 4 | 15.7 |
| 5 | 14.7 |
| ≥6 | 28.5 |
| Children under age 5 |  |
| 0 | 56.5 |
| 1 | 31.9 |
| ≥2 | 11.6 |
| Elderly over age 60 |  |
| 0 | 73.5 |
| 1 | 21.8 |
| ≥2 | 4.7 |
| Pregnant women |  |
| 0 | 97.0 |
| ≥1 | 3.0 |
| Wealth index |  |
| Poorest | 20.0 |
| Poorer | 20.0 |
| Middle | 20.0 |
| Richer | 20.0 |
| Richest | 20.0 |
