# Supplementary material for: Knowledge of COVID-19 symptoms, transmission, and prevention: Evidence from health and demographic surveillance in Southern Mozambique
Source: PLOS Glob Public Health. 2023 Nov 1;3(11):e0002532. doi: 10.1371/journal.pgph.0002532 (PMC10619866; doi:10.1371/journal.pgph.0002532)
Supplement: S3 Table — (DOCX) [file pgph.0002532.s008.docx]

| S3 Table. Had symptoms suggestive of COVID-19 since COVID-19 was first reported in Mozambique (N=2,465) | | | |
| --- | --- | --- | --- |
| Symptom | N | % (95% CI) |  |
| Flu-like symptoms | 1242 | 50.4 (48.4, 52.4) |  |
| Dry cough | 1182 | 48.0 (46.0, 49.9) |  |
| Headaches | 832 | 33.8 (31.9, 35.7) |  |
| Fever | 665 | 27.0 (25.2, 28.8) |  |
| Cough with sputum | 541 | 21.9 (20.3, 23.6) |  |
| Sore throat | 194 | 7.9 (6.9, 9.0) |  |
| Muscle pain | 186 | 7.5 (6.5, 8.7) |  |
| Difficulty breathing | 162 | 6.6 (5.6, 7.6) |  |
| Vomiting | 18 | 0.7 (0.4, 1.2) |  |
| Other | 78 | 3.2 (2.5, 4.0) |  |
| CI: confidence interval. Individuals could have experienced multiple symptoms, so the sum of the percentages exceed 100%. | | | |
