# Supplementary material for: Knowledge of COVID-19 symptoms, transmission, and prevention: Evidence from health and demographic surveillance in Southern Mozambique
Source: PLOS Glob Public Health. 2023 Nov 1;3(11):e0002532. doi: 10.1371/journal.pgph.0002532 (PMC10619866; doi:10.1371/journal.pgph.0002532)
Supplement: S4 Table — (DOCX) [file pgph.0002532.s009.docx]

| S4 Table. Sources of information about COVID-19, Mozambique (N=33,087), | | | |
| --- | --- | --- | --- |
| Source | N | % (95% CI) |  |
| TV | 14551 | 44.0 (43.4, 44.5) |  |
| Community leaders | 11980 | 36.2 (35.7, 36.7) |  |
| Radio | 11134 | 33.7 (33.1, 34.2) |  |
| Hospital | 10518 | 31.8 (31.3, 32.3) |  |
| SMS/WhatsApp | 6748 | 20.4 (20.0, 20.8) |  |
| CI: confidence interval | | | |
