# Supplementary material for: Knowledge of COVID-19 symptoms, transmission, and prevention: Evidence from health and demographic surveillance in Southern Mozambique
Source: PLOS Glob Public Health. 2023 Nov 1;3(11):e0002532. doi: 10.1371/journal.pgph.0002532 (PMC10619866; doi:10.1371/journal.pgph.0002532)
Supplement: S5 Table — (DOCX) [file pgph.0002532.s010.docx]

| **S5 Table**. Mediation of sources of COVID-19 information on the association between educational attainment and knowledge of symptoms index^a^ derived from principal components analysis, Mozambique, April 2021 – February 2022 (N=33,087) | | | | | |
| --- | --- | --- | --- | --- | --- |
|  |  | Controlled direct effect | Natural indirect effect | Total effect |  |
| Education | Characteristic | Estimate (95% CI) | Estimate (95% CI) | Estimate (95% CI) | Proportion mediated |
| Higher | TV | 1.15 (1.02, 1.28) | 0.41 (0.38, 0.45) | 1.35 (1.22, 1.48) | 0.31 |
| Technical | TV | 1.03 (0.92, 1.17) | 0.36 (0.33, 0.39) | 1.21 (1.10, 1.34) | 0.30 |
| Secondary | TV | 0.66 (0.63, 0.70) | 0.22 (0.20, 0.23) | 0.79 (0.76, 0.82) | 0.27 |
| Primary | TV | 0.31 (0.28, 0.34) | 0.07 (0.06, 0.07) | 0.36 (0.34, 0.39) | 0.19 |
| Higher | Hospital | 1.35 (1.23, 1.49) | -0.01 (-0.02, -0.01) | 1.35 (1.22, 1.48) | -0.01 |
| Technical | Hospital | 1.22 (1.10, 1.35) | -0.01 (-0.02, -0.01) | 1.21 (1.10, 1.34) | -0.01 |
| Secondary | Hospital | 0.79 (0.76, 0.83) | -0.01 (-0.01, -0.01) | 0.79 (0.76, 0.82) | -0.01 |
| Primary | Hospital | 0.36 (0.34, 0.39) | -0.01 (-0.01, 0.00) | 0.36 (0.34, 0.39) | -0.01 |
| Higher | Radio | 1.32 (1.19, 1.45) | 0.07 (0.05, 0.10) | 1.35 (1.22, 1.48) | 0.05 |
| Technical | Radio | 1.19 (1.07, 1.32) | 0.06 (0.04, 0.09) | 1.21 (1.10, 1.34) | 0.05 |
| Secondary | Radio | 0.77 (0.74, 0.80) | 0.04 (0.03, 0.05) | 0.79 (0.76, 0.82) | 0.05 |
| Primary | Radio | 0.34 (0.31, 0.37) | 0.03 (0.03, 0.04) | 0.36 (0.34, 0.39) | 0.09 |
| Higher | SMS/WhatsApp | 1.22 (1.09, 1.35) | 0.29 (0.26, 0.34) | 1.35 (1.22, 1.48) | 0.22 |
| Technical | SMS/WhatsApp | 1.09 (0.97, 1.22) | 0.27 (0.23, 0.31) | 1.21 (1.10, 1.34) | 0.22 |
| Secondary | SMS/WhatsApp | 0.71 (0.68, 0.75) | 0.14 (0.13, 0.15) | 0.79 (0.76, 0.82) | 0.18 |
| Primary | SMS/WhatsApp | 0.34 (0.31, 0.37) | 0.03 (0.03, 0.04) | 0.36 (0.34, 0.39) | 0.09 |
| Higher | Community leaders | 1.38 (1.25, 1.50) | -0.07 (-0.09, -0.05) | 1.35 (1.22, 1.48) | -0.05 |
| Technical | Community leaders | 1.25 (1.14, 1.38) | -0.10 (-0.11, -0.08) | 1.21 (1.10, 1.34) | -0.08 |
| Secondary | Community leaders | 0.82 (0.79, 0.86) | -0.07 (-0.08, -0.06) | 0.79 (0.76, 0.82) | -0.09 |
| Primary | Community leaders | 0.38 (0.35, 0.41) | -0.02 (-0.03, -0.02) | 0.36 (0.34, 0.39) | -0.07 |

^a^ Knowledge of symptoms index included: difficulty breathing, dry cough, fever, headaches, muscle pain, and sore throat
