# Supplementary material for: Knowledge of COVID-19 symptoms, transmission, and prevention: Evidence from health and demographic surveillance in Southern Mozambique
Source: PLOS Glob Public Health. 2023 Nov 1;3(11):e0002532. doi: 10.1371/journal.pgph.0002532 (PMC10619866; doi:10.1371/journal.pgph.0002532)
Supplement: S6 Table — (DOCX) [file pgph.0002532.s011.docx]

| **S6 Table**. Mediation of sources of COVID-19 information on the association between educational attainment and knowledge of transmission index^a^ derived from principal components analysis, Mozambique, April 2021 – February 2022 (N=33,087) | | | | | |
| --- | --- | --- | --- | --- | --- |
|  |  | Controlled direct effect | Natural indirect effect | Total effect |  |
| Education | Characteristic | Estimate (95% CI) | Estimate (95% CI) | Estimate (95% CI) | Proportion mediated |
| Higher | TV | 1.21 (1.07, 1.34) | 0.44 (0.40, 0.49) | 1.37 (1.23, 1.51) | 0.32 |
| Technical | TV | 0.97 (0.85, 1.10) | 0.35 (0.32, 0.39) | 1.12 (1.00, 1.25) | 0.32 |
| Secondary | TV | 0.62 (0.59, 0.66) | 0.21 (0.20, 0.22) | 0.73 (0.70, 0.76) | 0.29 |
| Primary | TV | 0.31 (0.29, 0.34) | 0.06 (0.06, 0.07) | 0.35 (0.33, 0.38) | 0.18 |
| Higher | Hospital | 1.35 (1.25, 1.52) | -0.05 (-0.08, -0.03) | 1.37 (1.23, 1.51) | -0.04 |
| Technical | Hospital | 1.13 (1.02, 1.27) | -0.05 (-0.07, -0.03) | 1.12 (1.00, 1.25) | -0.05 |
| Secondary | Hospital | 0.75 (0.72, 0.78) | -0.04 (-0.05, -0.03) | 0.73 (0.70, 0.76) | -0.05 |
| Primary | Hospital | 0.36 (0.33, 0.38) | -0.01 (-0.01, 0.00) | 0.35 (0.33, 0.38) | -0.02 |
| Higher | Radio | 1.34 (1.20, 1.48) | 0.11 (0.07, 0.15) | 1.37 (1.23, 1.51) | 0.08 |
| Technical | Radio | 1.09 (0.97, 1.22) | 0.09 (0.06, 0.12) | 1.12 (1.00, 1.25) | 0.08 |
| Secondary | Radio | 0.71 (0.68, 0.74) | 0.05 (0.04, 0.06) | 0.73 (0.70, 0.76) | 0.07 |
| Primary | Radio | 0.33 (0.30, 0.36) | 0.04 (0.04, 0.05) | 0.35 (0.33, 0.38) | 0.12 |
| Higher | SMS/WhatsApp | 1.21 (1.07, 1.35) | 0.43 (0.37, 0.49) | 1.37 (1.22, 1.51) | 0.31 |
| Technical | SMS/WhatsApp | 0.96 (0.85, 1.10) | 0.36 (0.32, 0.41) | 1.12 (1.00, 1.26) | 0.33 |
| Secondary | SMS/WhatsApp | 0.64 (0.60, 0.67) | 0.19 (0.18, 0.20) | 0.73 (0.70, 0.76) | 0.26 |
| Primary | SMS/WhatsApp | 0.33 (0.30, 0.38) | 0.05 (0.04, 0.05) | 0.35 (0.33, 0.38) | 0.13 |
| Higher | Community leaders | 1.39 (1.26, 1.53) | -0.10 (-0.12, -0.07) | 1.37 (1.23, 1.51) | -0.08 |
| Technical | Community leaders | 1.15 (1.04, 1.29) | -0.12 (-0.15, -0.10) | 1.12 (1.00, 1.25) | -0.11 |
| Secondary | Community leaders | 0.77 (0.74, 0.80) | -0.09 (-0.10, -0.08) | 0.73 (0.70, 0.76) | -0.12 |
| Primary | Community leaders | 0.37 (0.35, 0.40) | -0.03 (-0.03, -0.02) | 0.35 (0.33, 0.38) | -0.08 |

^a^ Knowledge of transmission index included: droplets from an infected person, hugging an infected person, kissing an infected person, touching a fomite, touching an infected person, touching an infected person’s hands, touching your eyes or nose, and touching your mouth
