# Supplementary material for: Knowledge of COVID-19 symptoms, transmission, and prevention: Evidence from health and demographic surveillance in Southern Mozambique
Source: PLOS Glob Public Health. 2023 Nov 1;3(11):e0002532. doi: 10.1371/journal.pgph.0002532 (PMC10619866; doi:10.1371/journal.pgph.0002532)
Supplement: S7 Table — (DOCX) [file pgph.0002532.s012.docx]

| **S7 Table**. Mediation of sources of COVID-19 information on the association between educational attainment and knowledge of prevention index^a^ derived from principal components analysis, Mozambique, April 2021 – February 2022 (N=33,087) | | | | | |
| --- | --- | --- | --- | --- | --- |
|  |  | Controlled direct effect | Natural indirect effect | Total effect |  |
| Education | Characteristic | Estimate (95% CI) | Estimate (95% CI) | Estimate (95% CI) | Proportion mediated |
| Higher | TV | 0.85 (0.74, 0.97) | 0.37 (0.33, 0.41) | 0.97 (0.86, 1.11) | 0.38 |
| Technical | TV | 0.72 (0.60, 0.84) | 0.31 (0.27, 0.35) | 0.83 (0.72, 0.95) | 0.37 |
| Secondary | TV | 0.42 (0.39, 0.45) | 0.17 (0.16, 0.18) | 0.50 (0.48, 0.53) | 0.34 |
| Primary | TV | 0.17 (0.15, 0.19) | 0.05 (0.04, 0.05) | 0.20 (0.18, 0.22) | 0.23 |
| Higher | Hospital | 0.99 (0.88, 1.11) | -0.08 (-0.12, -0.05) | 0.97 (0.86, 1.11) | -0.08 |
| Technical | Hospital | 0.86 (0.75, 0.96) | -0.09 (-0.12, -0.05) | 0.83 (0.73, 0.94) | -0.10 |
| Secondary | Hospital | 0.52 (0.50, 0.55) | -0.06 (-0.07, -0.05) | 0.50 (0.48, 0.53) | -0.12 |
| Primary | Hospital | 0.21 (0.19, 0.22) | -0.01 (-0.01, 0.00) | 0.20 (0.18, 0.22) | -0.04 |
| Higher | Radio | 0.94 (0.83, 1.07) | 0.11 (0.07, 0.14) | 0.97 (0.86, 1.10) | 0.11 |
| Technical | Radio | 0.81 (0.70, 0.92) | 0.09 (0.06, 0.12) | 0.83 (0.72, 0.94) | 0.11 |
| Secondary | Radio | 0.48 (0.46, 0.51) | 0.05 (0.04, 0.06) | 0.50 (0.48, 0.53) | 0.10 |
| Primary | Radio | 0.18 (0.16, 0.20) | 0.04 (0.03, 0.04) | 0.20 (0.18, 0.22) | 0.18 |
| Higher | SMS/WhatsApp | 0.84 (0.73, 0.96) | 0.39 (0.35, 0.45) | 0.97 (0.86, 1.11) | 0.40 |
| Technical | SMS/WhatsApp | 0.70 (0.60, 0.82) | 0.34 (0.29, 0.38) | 0.83 (0.72, 0.94) | 0.41 |
| Secondary | SMS/WhatsApp | 0.42 (0.39, 0.45) | 0.17 (0.16, 0.18) | 0.50 (0.47, 0.53) | 0.33 |
| Primary | SMS/WhatsApp | 0.18 (0.16, 0.20) | 0.04 (0.03, 0.04) | 0.20 (0.18, 0.22) | 0.18 |
| Higher | Community leaders | 1.01 (0.89, 1.13) | -0.15 (-0.19, -0.12) | 0.97 (0.86, 1.10) | -0.15 |
| Technical | Community leaders | 0.88 (0.77, 0.99) | -0.20 (-0.23, -0.16) | 0.83 (0.73, 0.95) | -0.24 |
| Secondary | Community leaders | 0.54 (0.52, 0.57) | -0.13 (-0.14, -0.12) | 0.50 (0.48, 0.53) | -0.26 |
| Primary | Community leaders | 0.22 (0.21, 0.24) | -0.04 (-0.04, -0.03) | 0.20 (0.19, 0.22) | -0.18 |

^a^ Knowledge of prevention index included: avoid crowded places, touching eyes, touching mouth, touching nose, or traveling; social distancing; quarantine; and wash hands with alcohol
