# Supplementary material for: Knowledge of COVID-19 symptoms, transmission, and prevention: Evidence from health and demographic surveillance in Southern Mozambique
Source: PLOS Glob Public Health. 2023 Nov 1;3(11):e0002532. doi: 10.1371/journal.pgph.0002532 (PMC10619866; doi:10.1371/journal.pgph.0002532)
Supplement: S8 Table — Generalized variance inflation factors of independent variables for analysis of associations between demographic characteristics and knowledge of COVID-19, Mozambique, April 2021 –February 2022 (N = 33,087). (DOCX) [file pgph.0002532.s013.docx]

| S8 Table. Generalized variance inflation factors of independent variables for analysis of associations between demographic characteristics and knowledge of COVID-19, Mozambique, April 2021 – February 2022 (N=33,087). | |
| --- | --- |
| Variable | **Generalized variance inflation factor** |
| Education | 1.097399 |
| Age group | 1.170266 |
| Occupation | 1.046221 |
| Sex | 1.123760 |
| Religion | 1.024557 |
| Language | 1.012741 |
| Had COVID-19 symptoms | 1.021757 |
| Marital status | 1.106240 |
| Household size | 1.057320 |
| Adults over 60 | 1.158546 |
| Children under 5 | 1.117738 |
| Pregnant women | 1.008662 |
| Wealth index | 1.072566 |
| Community leaders | 1.121468 |
| Hospital | 1.099873 |
| Radio | 1.040220 |
| SMS/WhatsApp | 1.103482 |
| TV | 1.292671 |
